# Clonal pattern dynamics in tumor: the concept of cancer stem cells

**DOI:** 10.1038/s41598-019-51575-1

**Published:** 2019-10-30

**Authors:** Fabrizio Olmeda, Martine Ben Amar

**Affiliations:** 1 Max Planck Institute for the Physics of Complex Systems, Nöthnitzer Str. 38, D-01187 Dresden, Germany; 2 Laboratoire de Physique de l’Ecole normale supérieure, ENS, Université PSL, CNRS, Sorbonne Université, Université de Paris, F-75005 Paris, France; 3 Institut Universitaire de Cancérologie, Faculté de médecine, Sorbonne Université, 91 Bd de l’Hôpital, 75013 Paris, France

**Keywords:** Biophysics, Mathematics and computing, Physics

## Abstract

We present a multiphase model for solid tumor initiation and progression focusing on the properties of cancer stem cells (CSC). CSCs are a small and singular cell sub-population having outstanding capacities: high proliferation rate, self-renewal and extreme therapy resistance. Our model takes all these factors into account under a recent perspective: the possibility of phenotype switching of differentiated cancer cells (DC) to the stem cell state, mediated by chemical activators. This plasticity of cancerous cells complicates the complete eradication of CSCs and the tumor suppression. The model in itself requires a sophisticated treatment of population dynamics driven by chemical factors. We analytically demonstrate that the rather important number of parameters, inherent to any biological complexity, is reduced to three pivotal quantities.Three fixed points guide the dynamics, and two of them may lead to an optimistic issue, predicting either a control of the cancerous cell population or a complete eradication. The space environment, critical for the tumor outcome, is introduced via a density formalism. Disordered patterns are obtained inside a stable growing contour driven by the CSC. Somewhat surprisingly, despite the patterning instability, the contour maintains its circular shape but ceases to grow for a typical size independently of segregation patterns or obstacles located inside.

## Introduction

Discrete^1–3^ and continuous^4^ models have been proposed recently to study solid tumor progression within the cancer stem cells (CSCs) hypothesis. According to this concept^5^, CSCs are a small subpopulation within the tumor that are the main cause for its progression. These cells have high proliferation rates, self-renewal and quasi-immortality capacities^6,7^. Consequently, they are very difficult to detect and to eradicate. The cancer stem cell hypothesis has been matter of strong debates during the last forty years^8^. Nevertheless it explains several biological evidences such as the relapse of tumor post-surgery and treatments^9^ or the ability to generate tumors in xenotransplantation^10^. Nowadays, surface biomarkers, in charge of identification and detection of these cells, are more and more evaluated for different classes of cancer which allows new therapeutics in clinical^11^ but also experimental measurements of their physical properties^12^.

The CSC model^13^ enters into a more general class, usually referred as hierarchical models where cancer cells originate from a precursor which divides either symmetrically giving two identical CSCs or two DCs (differentiated cancer cells) or asymmetrically (one CSC and one DC). In these models, only the complete extinction of the CSC population eradicates the tumor. The stochastic model, instead, considers the possibility that every cell can be a tumor-initiating one and so the best eradication strategy becomes a more complex issue. Our work lies between both concepts: indeed, as stated by Plaks *et al*.^14^, this distinction is often a false dichotomy because of the phenotype plasticity of cancerous cells^15^ which allows to recover the stem traits by dedifferentiation. The same feature also occurs with healthy differentiated cells which are known to gain a stem state under traumatic conditions, but not only, and this plasticity process seems much more common than previously believed^8^. Within these ideas, we propose a rather simple dynamical tumor initiation model in the continuous limit, for a mixed population, composed of 3 subpopulations: cancer stem cells (CSCs), differentiated cancer cells (DCs) and all other cells that a tumor harbors such as quiescent, dead or immune cells. Each cell population, represented by its density, enters a nonlinear dynamical system based on proliferation rates controlled by chemical pathways and physical interactions. We demonstrate that the dynamics is governed by the existence and stability of 3 fixed points showing the possibility of full tumor extinction, but also of over-shooting dynamics after a period of remission or relapse of the tumor post surgery.These features, observed in practice, are not commonly recovered in cancer growth modeling^16,17^ when biological parameters are fixed.

According to activator and inhibitor concentrations which control the growth rate or the phenotypic changes^6,8^, a phase-diagram is presented,  which shows the exchange of stability as a function of a very limited number (3) of pivotal parameters. Focusing on the conditions for final extinction by a proper selection of the parameters is indeed a crucial goal for efficient therapeutic strategies^9^, giving hope to bypass the cell plasticity.

We also aim to determine the spatial cellular repartition during tumor progression. From a physical viewpoint, we provide a methodology for the study of ternary mixtures which have been poorly investigated, especially for free boundary growing domains^18–20^. Involving cell-cell interaction and solid-fluid friction, the mechanics is based on variational analysis which leads to a set of coupled partial-differential equations (PDE). The simulations tackle space inhomogeneity, possible obstacles, applied exterior stresses and the emergence of different tumors in the same neighborhood, then mimicking more closely the tumoral micro-environement and giving a most realistic picture of growing tumors in patients. In addition, the basic concepts are not limited to cancer research and may also apply to other ecologic^21^ or societal models^22,23^, where a colony expands thanks to a very active and aggressive sub-population.

## The Theoretical Model

**Figure 1:**
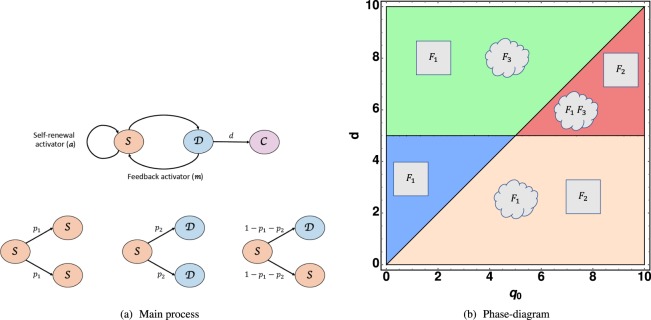
(**a**) Schematic presentation of the model corresponding to the set of equations Eq. (1) (**b**) Phase diagram for existence and stability of fixed points in the plane of dimensionless parameters *d*,*q*_0_. Regions are separated by *d* = *q*_0_ and by *d* = *ψα*/*β*. The range *γ*/*α* > *m*_0_ has been selected. Stable fixed points are represented by a square, saddle fixed points by a speech bubble. In the plastic region, *d* < *q*_0_, the value of the parameters are intimately related, *m*_2_ being always close to *m*_0_ indicating an easy phenotypic switching and so plasticity. Close to ${{\mathscr{ {\mathcal F} }}}_{2}$, tumor cells are then in the highest plastic state. In the following, Table 2 classifies all the fixed points in each region of the phase diagram.

We restrict ourselves to 3 kinds of cells with the same mass density: cancer stem cells (CSC), differentiated cancer cells (DC) and all other cells (C) or constituents such as interstitial fluids. In a closed environment, the concentrations *S*, for CSC, *D* for DC and *C* for inert constituents satisfy the relation *S* + *D* + *C* = 1. We simplify the proliferation process of the CSCs, eliminating intermediate progenitors^24,25^. The main characteristic of stem-ness is the fact of symmetric or asymmetric divisions leading to clones into the hierarchy that we describe in terms of probabilities (see Fig. (1a)). CSCs divide either symmetrically, giving two CSCs with probability *p*_1_ or two DCs with probability *p*_2_, or asymmetrically with probability 1 − *p*_1_ − *p*_2_. Then the probability to gain 2 CSCs per division reads 2*p* = (1 + *p*_1_ − *p*_2_) while it becomes 2(1 − *p*) = 1 − *p*_1_ + *p*_2_ for DC cells, see Fig. (1a). The mitosis of CSC is simultaneously controlled chemically by an activator **a** and an inhibitor **i**.**a** is produced by the CSCs (via the Wnt-*β* pathways^26^), and **i** is produced by the DCs, aiming to prevent the uncontrolled growth of *S*. Consequently, the probability 2*p* will increase with the activator concentration **a** saturating for large **a** values while the inhibitor **i** will decrease the *p* value as *D* increases. However, when *S* becomes exceptionally low, (falling below a tiny *S*_0_ value), mitosis alone is not sufficient for re-population of the CSC community. There is strong experimental evidence that a feedback exists, originating from phenotypic changes of DC cells switching back to the CSC state. Also called cancer cell plasticity, this process is governed by an activator **m** which can be a network of miniRNA^27^ for example. Surprisingly, in a sorted population with only DCs, where this process happens, it does not lead to an over-shoot of the CSC population, so the effect is weak but relatively sharp around the *S*_0_ value.

In summary, the dynamics of cell population and of the chemicals is governed by a set of 4 coupled differential equations with smooth variations^4^, except for the activator m^1^ which varies sharply around the value *S* ~ *S*_0_. All these assumptions explain the following set of equations:

1 $$\{\begin{array}{ccc}\frac{dS}{dt} & = & {\Gamma }_{S}=(2p(D,a)-1)S+q(m)D\\ \frac{dD}{dt} & = & {\Gamma }_{D}=2(1-p(D,a))S-(d+q(m))D\\ \frac{da}{dt} & = & {\Gamma }_{a}=a(\beta S\frac{a}{1+a}-\alpha )\\ \frac{dm}{dt} & = & {\Gamma }_{m}=\gamma {e}^{-S/{S}_{0}}-\alpha m\end{array}$$

where *p*(*D*, *a*) and *q*(*m*) satisfy:

2 $$\{\begin{array}{ccc}p(D,a) & = & \frac{\eta a}{(1+\eta a)(1+\psi D)}\\ q(m) & = & \frac{{q}_{0}}{2}(1+\,\tanh (\frac{m-{m}_{0}}{\sigma }))\end{array}$$

At this stage, the spatial variation is ignored and the time unit *t*_0_ has been chosen as the inverse of the mitotic rate of the CSCs. CSCs are considered as immortal, as opposed to DCs and to the chemicals **a** or **m**,  so no death rate is added for *S*. According to the second equation of Eq. (1), the DC proliferation and death are included in the single parameter *d*. Excessive growth of *D* leads to the disappearance of CSCs, the so-called Allee effect, demonstrated by Konstorum *et al*.^4^ and to an optimistic issue for tumor elimination. Unfortunately, as CSCs tend to disappear, the de-differentiation (or plasticity) occurs from DC to CSC, controlled by **m** as shown in Fig. (1). Then, the Allee effect may be compromised by the re-population of CSCs, and ultimately of DCs, which happens for **m** greater than a typical value *m*_0_. To ensure a sharp variation of *q*(*m*), the parameter *σ* which enters the last equation of Eq. (2) is a tiny quantity and a real feedback DCs versus CSCs is possible if *γ*/*α* > *m*_0_. In practice, *in vivo* or *in vitro*, the population of CSCs is always a tiny fraction of the entire tumor population (few per cents). When *S* is below this tiny value, the activator **m** starts to grow as *q*(*m*), leading to an increase of *S*. Notice that the time-scale for de-differentiation *q*_0_, Eq. (2), has to be compared to the symmetric mitotic rate but the issue of this dynamical system depends on the comparison of *q*_0_ with *d* (the time death rate of DCs) and with *α* (time elimination rate of both chemicals **a** and **m** which are chosen equal for simplification). As in^4^, the dynamics of **a** results from the positive competition between the activator itself, the CSC’s population *S* and the spontaneous degradation rate *α*. To conclude, in our model, the selected biological ingredients of the CSCs hypothesis leads to a dynamical system of population of fourth order with a minimal set of 8 parameters, two of them having small values as *S*_0_ and *σ*. As demonstrated in the next section and suggested by the phase diagram in Fig.(1b), only 3 parameters really control the full dynamics and the tumor progression.

## Dynamics: Fixed Points, Stability, Basin of Attraction

### Existence of fixed points

The strategy of population dynamics consists in the search of fixed points, achieved by cancelling all time derivatives in Eq. (1). Followed by stability analysis, this part does not present any difficulty and details can be found in the Appendix (A). Here we only focus on the main results. Fixed points represent static and homogeneous solutions of the physical system, which is  assumed to be spatially infinite. Defined by the 4 concentrations: ${ {\mathcal F} }_{i}$ = {*S*_*i*_, *D*_*i*_, *a*_*i*_, *m*_*i*_}, they share in common 2 relationships:

3 $${m}_{i}=\frac{\gamma }{\alpha }\exp (-\,{S}_{i}/{S}_{0})\,{\rm{and}}\,{S}_{i}=d{D}_{i}$$

so only *S*_*i*_ and *a*_*i*_ must be determined since *S*_*i*_ determines both *D*_*i*_ and *m*_*i*_. A calculation with *a*_*i*_ = 0, so *p* = 0 leads to 2 fixed points: *S*_1_ = 0 or ${S}_{2}=-\,{S}_{0}\,\log (\frac{\alpha }{\gamma }{m}_{2})$. The first fixed point accepts arbitrary *m*_1_ value, ${ {\mathcal F} }_{1}$ = {0, 0, 0, *m*_1_} and corresponds to the full tumor extinction. For the second choice, *m*_2_ satisfies:

4 $${m}_{2}={m}_{0}+\sigma {\tanh }^{-1}(\frac{2d}{{q}_{0}}-1),\,{\rm{and}}\,0 < d < {q}_{0},$$

according to Eq. (1). In other words, this second fixed point only exists under two conditions: first, there is no self-proliferation of the DCs and second, cancerous cell plasticity occurs. The first condition then implies that the *D* cells are in an homeostatic state. In practice, both *S* and *D* must remain smaller than 1 just like their sum, but more importantly *S* and *D* must remain positive leading to a new inequality *m*_2_ < *γ*/*α*. Then, the existence of the second fixed point imposes the following inequalities of parameters: 0 < *d* < *q*_0_ and *m*_2_ < *γ*/*α*.

Considering now *a*_*i*_ ≠ 0, the third equation of Eq. (1) gives *S*_3_ = *α*(1 + *a*_3_)/(*βa*_3_), so ${m}_{3}=\gamma /a{e}^{-{S}_{3}/{S}_{0}}$ and finally *D*_3_ = *S*_3_/*d*, see Eqs (16, 25). However, the first equation must be verified leading to (2*p*(*D*_3_,*a*_3_) − 1)*d* + *q* = 0 so *p* ~ 1/2, for small *S*_0_ and *σ*. An algebraic equation of second order for *a*_3_ gives a positive solution if *ψ* < *d*(*β*/*α*) (see Appendix C). Then the existence of this third fixed point requires an efficient but not too strong retro-action originating from the DCs.

A summary of these conclusions can be found in the phase-diagram in Fig. (1b) which shows that 2 fixed points always exist, ${ {\mathcal F} }_{1}$ and ${ {\mathcal F} }_{3}$, contrary to ${ {\mathcal F} }_{2}$ which only exists if 0 < *d* < *q*_0_. Independently of the parameter restrictions, these fixed points are potentially relevant for tumor growth if they are stable with a finite basin of attraction. This is the reason why we consider now the dynamics of small perturbations, described by the linearization of the right-hand-side of Eq. (1).

### Stability of the fixed points

**Figure 2:**
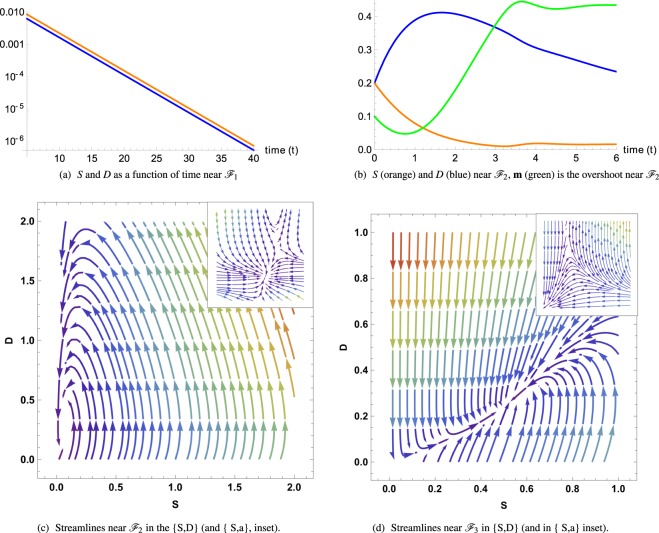
(**a,b**) Cancer cell *S* and *D* evolution near fixed points ${ {\mathcal F} }_{1}$ and ${ {\mathcal F} }_{2}$. (**c,d**) Streamlines determine the direction of attraction or repulsion near the fixed points for the tumor cells and the chemicals. Due to the high dimensionality of the system we project the dynamics along two directions, while keeping the other two constant. In (**c,d**) A complex pair of eigenvalues with negative real part determines a spiral point in the {S, D} plane. Differently, the other two eigenvalues are real and negative, making the point ${ {\mathcal F} }_{2}$ globally stable (**c**). (**c,d**) arrows determine the direction of attraction or repulsion near the fixed points for the tumor cells and the chemicals and the colors, increasing from purple to red, are the relative norms of the vector fields. Due to the high dimensionality of the system, we project the dynamics along two directions, while keeping the other two constant.
- ${ {\mathcal F} }_{2}$ = (*S*_2_, *S*_2_/*d*, 0, *m*_2_) means a low population for both CSCs and DCs. It is always stable when it exists (*d* < *q*_0_) below the first bisector in a phase-diagram Fig.(1b). In Figure (2b,c), from an initial set of parameters inside the basin of attraction of ${ {\mathcal F} }_{2}$, the streamlines and the dynamics of both *S*(*t*) and *D*(*t*) are presented.  The concentrations exhibit an over-shooting behavior before reaching the equilibrium values (see Fig. (2b)) and that the streamlines are disordered in Fig. (2c).
- The dynamics of ${ {\mathcal F} }_{3}$ is more complex and requires appropriate approximations. Assuming *ψ* < *dβ*/*α*, it is at least a saddle point, possibly an unstable point, see Fig. (2d) where it is a saddle fixed point.

At linear order, rewritten in matrix form, it reads:

5 $$\frac{d}{dt}{({\rm{\delta }}{ {\mathcal F} }_{i})}^{T}=J({ {\mathcal F} }_{i}){({\rm{\delta }}{ {\mathcal F} }_{i})}^{T}$$

where (δ${ {\mathcal F} }_{i}$)^*T*^ is the transpose of δ${ {\mathcal F} }_{i}$ and *J*(${ {\mathcal F} }_{i}$) is the Jacobian calculated for each fixed point (see the Appendix A). The stability is governed by the sign of the real part *λ*^*R*^ of the eigenvalues *λ*_*i*_ of the Jacobian matrix: it is stable if all *λ*^*R*^ values are negative (locally an attractor), unstable if all eigenvalues are positive (a repeller), and saddle in the general case. Each fixed point must be treated separately with technical aspects explained in the Appendix B,C and the stability results are functions of the model parameters which are summarized here:

- ${ {\mathcal F} }_{1}$ = (0, 0, 0, *m*_1_), which leads to the full tumor extinction, is stable and attractive if *d* > *q*(*m*_1_).  If the feedback of DCs versus CSCs is not too strong in comparison with the death rate of *D*. Since *q*(*m*_1_) < *q*_0_, the stability condition for ${ {\mathcal F} }_{1}$ is simply *d* > *q*_0_ which means *d* above the first bisector in the phase-diagram Fig. (1b). In Figure (2a), the monotonous decays of both *S*(*t*) and *D*(*t*) from an initial set parameters inside the basin of attraction of ${ {\mathcal F} }_{1}$ are outlined.

To conclude, among all parameters of the model, only *d* and *q*_0_ play a pivotal role. It exists always a stable fixed point with low cancerous cell concentration: either ${ {\mathcal F} }_{1}$ or ${ {\mathcal F} }_{2}$. When ${ {\mathcal F} }_{2}$ appears, for *d* < *q*_0_, ${ {\mathcal F} }_{1}$ becomes unstable. When ${ {\mathcal F} }_{3}$ exists, it is never stable. For a clinical perspective, to reach either ${ {\mathcal F} }_{1}$ or ${ {\mathcal F} }_{2}$ takes a great importance. However when the feedback and plasticity of DCs is too weak compared to their death rate, the emergence of ${ {\mathcal F} }_{3}$ may compromise these optimistic solutions. One can wonder if the scenario depicted here is maintained when the feedback parameter *q*_0_ becomes a time-dependent variable, *q*_0_ = *q*_0_(*t*) or if some chemical values as *a* or *m* become stochastic. This may occur for multiple reasons, the main coming from the genetic of cancerous cells, mutation after mutation. Another reason may be environmental factors, changing in the chemicals activity as drug for example. In the Appendix, D, we perform numerical simulations of the dynamical model, Eq. (1), with a time-dependent *q*_0_(*t*) and we give preliminary results on stochastic  effects.

#### Strategy for tumor regression

The previous analysis shows the dramatic role of two quantities: *d* and *q*(*m*) representing respectively the DCs proliferation rate (*d* < 0) or the DCs death rate (*d* > 0) and *q*(*m*) the DCs dedifferentiation rate. A parameter *d* < 0 (by absorption of nutrients, for example) induces an exponential growth in both populations of CSCs and DCs.  Even for *d* > 0, (by senescence or death via drug treatments), it may not be sufficient even if the death rate *d* is stronger than the plasticity represented by *q*(*m*). In other words, if *d* > *q*(*m*) holds, it will not imply automatically a regression in all cases due to the vicinity of the third repulsive fixed point ${ {\mathcal F} }_{3}$.

The ideal case would be to remain in the blue region of the diagram, (Fig.(1b)), where there exists a unique stable fixed point ${ {\mathcal F} }_{1}$, insisting that, in this case, *q*(*m*_1_) < *d* < *ψα*/*β*. The most obvious strategy will consist in reducing *q*(*m*) as much as possible so that the first inequality becomes easier to reach. Since *m*_1_ = *γ*/*α* (see Eq. (3)) if *m*_1_ < *m*_0_, then *q*(*m*_1_) ~ 0, the first inequality is checked and there is hope to reach complete extinction of the tumor by eradicating both CSCs and DCs. Notice that, it requires also the second inequality to be verified. In this case, one should control the degradation rate *α* of the activators which plays a crucial role. Increasing *α* will be the best way to lead the tumor to extinction. Moreover, controlling the aggressiveness of the self-renewal activator via the parameter *β* as well as the inhibitory feedback of the DCs via *ψ* (Eq. (2) makes this situation more likely to occur (see Fig. (6a,b) in the Appendix D.1). The inset of Fig. (2c) representing the (*S*, *a*) subspace at fixed value *d* = 0 shows the small size of the basin of attraction, meaning that even at low levels of *S* and *a*, the tumor cannot undergo full tumor regression and differentiated cells may be produced again. Considering now *m*_0s_ and *m*_1_ fixed, varying *σ* will not affect too much *q*(*m*_1_) since *σ* only controls the smoothness of the function *q*(*m*).

Suppose now that *m*_1_ > *m*_0_, then *q*(*m*_1_) ~ *q*_0_. The worst scenario occurs when *d* < *q*_0_ because the stable attractive fixed point will be the second one ${ {\mathcal F} }_{2}$. What we would really like to do is to decrease as much as possible the value of *S*_2_ and *D*_2_, concentrations of CSCs and DCs respectively. But, *S*_2_ is of order *S*_0_, *D*_2_ is of order *S*_0_/*d*. It will be difficult to decrease these cancerous populations since they are dependent on biological parameters that we cannot control. Estimation of the time scale for proliferation of CSCs depends on the cancer origin. In^27^, the rate of cell division is about 1.8 month^−1^, and estimation of the de-differentiation factor is of order 0.6 per month. In pancreatic xenograft tumors, the rate of CSCs division is about 10 month^−1^ and the level of activator, normalized to 1 for healthy tissue, is about 4 for unsorted cancer cells, becoming 46 for selected and sorted CSCs^28^. Introducing these to Eq. (1), we derive *α*/*β* ~ 0.2 and *d* ~ 0.3. In Table 1, experimental results have been collected. Even if the biological literature on the subject is very rich these last years, it is  unfortunate that not so much quantitative physical or chemical informations are available.Finally in^29^, the inhibitor *i* has been identified explaining why patients with high level in lung tumor have a better  prognosis.

**Table 1:** Experimental data for various identified CSCs. The numerical parameter values for modeling take into account these data. Not all of them seem to be available in the literature.

*D*_*b*_ DC density at equilibrium, Eq. (9)	0.6−0.9	Reported in^55^
*τ* interphase friction, Eq. (8)	963−11571 mm^−2^ Pa day	^56, 57^
*χ* interstitial fluid pressure, Eq. (8)	1330 Pa	in skin carcinoma^55^
*α*_*D*_ = *χ*/*τ*, Eq. (9)	0.1 mm^2^ day^−1^	
Times unit *t*_0_	0.3−0.5 day	compatible with^5,27^
Length unit: $\sqrt{{t}_{0}\chi /\tau }$	0.1−0.3 mm	
Tumor expansion	~0.1−0.5 mm per day	compatible with^5,43^
*α*/*β*, Eq. (1)	0.1−0.3	estimated from data by^28^
*β*, Eq. (1)	0.15−1	estimated from data by^28^
*d*, Eq. (1)	~0.1−0.3 day −1.	from^5^
*D*_*a*_ or *D*_*m*_, Eq. (6)	0.001−0.01 mm^2^/day	from^55^

**Table 2:** Fixed points of the four regions of the phase diagram of Fig. (1b).

$$d > {q}_{0},d < \psi \alpha /\beta $$		
$${ {\mathcal F} }_{1}$$		
*λ*_1_ ∈ ${\mathbb{R}}$,*λ*_1_ < 0		
*λ*_2_ ∈ ${\mathbb{R}}$,*λ*_2_ < 0		
*λ*_3_ ∈ ${\mathbb{R}}$, *λ*_3_ < 0		
*λ*_4_ ∈ ${\mathbb{R}}$, *λ*_4_ < 0		
$$d < {q}_{0},d < \psi \alpha /\beta $$		
${ {\mathcal F} }_{1}$	${ {\mathcal F} }_{2}$	
*λ*_1_ ∈ ${\mathbb{R}}$, *λ*_1_ < 0	*λ*_1_ ∈ ${\mathbb{R}}$, *λ*_1_ < 0	
*λ*_2_ ∈ ${\mathbb{R}}$, *λ*_2_ < 0	*λ*_2_ ∈ ${\mathbb{R}}$, *λ*_2_ < 0	
*λ*_3_ ∈ ${\mathbb{R}}$, *λ*_3_ > 0	${\lambda }_{3}\in {\mathbb{C}},\Re [{\lambda }_{3}] < 0$	
*λ*_4_ ∈ ${\mathbb{R}}$, *λ*_4_ > 0	${\lambda }_{4}\in {\mathbb{C}},\Re [{\lambda }_{4}] < 0$	
$$d > {q}_{0},d > \psi \alpha /\beta $$		
${ {\mathcal F} }_{1}$	${ {\mathcal F} }_{3}$	
*λ*_1_ ∈ ${\mathbb{R}}$, *λ*_1_ < 0	*λ*_1_ ∈ ${\mathbb{R}}$, *λ*_1_ < 0	
*λ*_2_ ∈ ${\mathbb{R}}$, *λ*_2_ < 0	*λ*_2_ ∈ ${\mathbb{R}}$, *λ*_2_ > 0	
*λ*_3_ ∈ ${\mathbb{R}}$, *λ*_3_ < 0	${\lambda }_{3}\in {\mathbb{C}},{\rm{\Re }}[{\lambda }_{3}]\lessgtr 0$	
*λ*_4_ ∈ ${\mathbb{R}}$, *λ*_4_ < 0	${\lambda }_{4}\in {\mathbb{C}},{\rm{\Re }}[{\lambda }_{4}]\lessgtr 00$	
$$d < {q}_{0},d > \psi \alpha /\beta $$		
${ {\mathcal F} }_{1}$	${ {\mathcal F} }_{2}$	${ {\mathcal F} }_{3}$
*λ*_1_ ∈ ${\mathbb{R}}$, *λ*_1_ < 0	*λ*_1_ ∈ ${\mathbb{R}}$, *λ*_1_ < 0	*λ*_1_ ∈ ${\mathbb{R}}$, *λ*_1_ < 0
*λ*_2_ ∈ ${\mathbb{R}}$, *λ*_2_ < 0	*λ*_2_ ∈ ${\mathbb{R}}$, *λ*_2_ < 0	*λ*_2_ ∈ ${\mathbb{R}}$, *λ*_2_ > 0
*λ*_3_ ∈ ${\mathbb{R}}$, *λ*_3_ > 0	${\lambda }_{3}\in {\mathbb{C}},\Re [{\lambda }_{3}] < 0$	${\lambda }_{3}\in {\mathbb{C}},{\rm{\Re }}[{\lambda }_{3}]\lessgtr 0$
*λ*_4_ ∈ ${\mathbb{R}}$, *λ*_4_ > 0	${\lambda }_{4}\in {\mathbb{C}},\Re [{\lambda }_{4}] < 0$	${\lambda }_{4}\in {\mathbb{C}},{\rm{\Re }}[{\lambda }_{4}]\lessgtr 0$

Every fixed point is classified with respect to the eigenvalues of the corresponding Jacobian matrix. Every eigenvalue is fully determined but for ${ {\mathcal F} }_{3}$, the pair of complex conjugate eigenvalues may have either a positive or negative real part (in the table). This will not change the global stability, since an eigenvalue with positive real part *(λ*_2_ in the Table) always exists as demonstrated in the Appendix C. In the blue region (*d* > *q*_0_, *d* < *ψα*/*β*), only one fixed point exists with real and negative eigenvalues. Every initial concentration of cells and chemicals will then decay exponentially to ${ {\mathcal F} }_{1}$, which indicates a complete tumor regression. In the orange region (*d* < *q*_0_, *d* < *ψα*/*β*), the Jacobian near ${ {\mathcal F} }_{1}$ has two positive and real eigenvalues, leading to a long time exponential relaxation for arbitrary initial concentration to ${ {\mathcal F} }_{2}$. In the green region (*d* > *q*_0_, *d* > *ψα*/*β*), every initial concentration in the basin of attraction of ${ {\mathcal F} }_{1}$, will exponentially relax to full regression, whilst increasing if it lays outside the basin. A complete discussion about this scenario has been given in the main text (Section (Dynamics)). In the red region of Fig. 1, (*d* < *q*_0_, *d* > *ψα*/*β*), the scenario is very rich, due to the existence of all fixed points. The Jacobian of ${ {\mathcal F} }_{1}$ has two real and positive eigenvalues, making this point unstable. The tumor will never undergo regression. Instead, ${ {\mathcal F} }_{2}$ is globally stable; an initial concentration of chemicals and cells in the vicinity of ${ {\mathcal F} }_{2}$ will relax to a low population tumor. Differently from the orange region, the existence of ${ {\mathcal F} }_{3}$ restricts the basin of attraction of ${ {\mathcal F} }_{2}$, making the tumor less likely to relax to ${ {\mathcal F} }_{2}$.

Thus, the best strategy seems to target the chemical activators rather than the cancerous cells. Indeed, the presence of the cancer stem cells effectively drives the tumor, even in the optimistic case where we hopefully kill all the cancer cells, still CSCs will differentiate again and start a new cycle making the treatment very unlikely to succeed when the chemical activators are not controlled, (compare the two figures Fig. (6a,b) in the Appendix D.1 which shows how the basin of attraction of a stable point can be reduced). In medical centers nowadays, this strategy is commonly employed post-surgery to avoid the relapse of a new tumor at the same place of the primary one which has been withdrawn. Long-time drug administration over years aims at suppressing these activators.

The analysis of this model is very rich. However, it assumes an infinite geometry and the results must be confronted to the spatial structure of the host tissue. It is the reason why we now focus on the spatial evolution of a tumor seed, with initial conditions in the vicinity of the fixed point ${ {\mathcal F} }_{2}$.

## Spatio-Temporal Dynamics of Tumor Growth

Population dynamics modeling gives only a first overview of tumoro-genesis, far from the real complexity driven by cell-cell interactions, friction with other cells or with the stroma and physical perturbations due to obstacles, that are always present in the host organ. In addition, in many cancers, the primary tumor, once detected, is not unique and may be accompanied by smaller satellites. So the spatial component cannot be ignored as well. As shown in^30^, for inhomogeneous *in vivo* tumor, the density of each constituent varies locally, and the spatio-temporal behavior is completely different from the one predicted by a dynamical system. It is even worse when a spinodal decomposition^19^ occurs leading to a a phase separation of Cahn-Hilliard type inside the tumor^31,32^. Furthemore, the possible existence of niches, area of localization of *S* cells, introduces a new complexity that we will not consider here. They do not seem to be present in every tumor and are believed to be dependent on the patient’s age, as reported by Ferraro and Lo Clesio^33^. Besides, most of the physical characteristics of the cancerous niche such as their localization and density in the host tissue have not been reported even in recent review papers as^6,8,26^ which focus mostly on biological pathways.

Given the diversity of situations for human beings, a unique model cannot exist and the same strategy is adopted here for CSCs and DCs. Our dynamical system must be completed by adding the standard advective term $\nabla \cdot ({\varphi }_{i}{\overrightarrow{v}}_{i})$ in the left-hand-side of Eq. (1), ϕ_*i*_ representing either *S*, *D* or *a*, *m*. It reads:

6 $$\{\begin{array}{ccc}\frac{dS}{dt}={\Gamma }_{S}-\nabla \cdot S{\overrightarrow{v}}_{S} & {\rm{and}} & \frac{dD}{dt}={\Gamma }_{D}-\nabla \cdot D{\overrightarrow{v}}_{D}\\ \frac{da}{dt}={\Gamma }_{a}+{D}_{a}\Delta a\, & {\rm{and}} & \frac{dm}{dt}={\Gamma }_{m}+{D}_{m}\Delta m\end{array}$$

For the chemicals, **a** or **m**, we restrict on diffusion and ${\overrightarrow{v}}_{i}=-\,{D}_{i}\nabla {\varphi }_{i}/{\varphi }_{i}$. The average velocity of cells ${\overrightarrow{v}}_{i}$ will be derived from the Rayleighian variation, counter-part of the Lagrangian for highly dissipative systems^34,35^, taking into account the constraints of the Galilean Invariance: $\nabla (S{\overrightarrow{v}}_{S}+D{\overrightarrow{v}}_{D}+C{\overrightarrow{v}}_{C})=0$ in addition to *S* + *D* + *C* = 1 (see the Appendix E). Then, cell velocities result from proliferation and mechanical forces. CSCs being much less numerous and more individualist with a propensity to escape and make metastasis, we neglect their energy of cohesion. Thus, the free energy of the cell mixing *E* is dominated by the DCs^36^, and we only consider the mixing energy of DCs:

7 $${\mathscr{E}}=\mathop{\int }\limits_{\Omega (t)}\,(\chi (D)+\frac{{\varepsilon }^{2}}{2}{(\nabla D)}^{2})d\Omega ,$$

Ω(*t*) defining the tumor domain in expansion, *χ* only depends on the density *D*, the last term being a penalty for sharp transitions^19,35,37^. At the border, the micro-environment acts as a hydrostatic pressure. Little is known on cell-cell interaction of CSCs with DCs, but the mixing energy is scaled by the square of cell densities giving a net advantage to the DCs, responsible for the tumor cohesion. The cellular elasticity is discarded: recent AFM^38,39^ or nano-indentation experiments^40^ indicate that cancerous cells are less stiff than their healthy counterparts by 70% or 80%, due to an alteration and regression of their cytoskeleton. Since inertia is negligible, viscous dissipation controls the dynamics which is determined by a variational process acting on the Rayleighian (for technical details see Appendix E). Taking into account the principle of extremum of this quantity with the incompressibility constraint gives the following velocities for ${\overrightarrow{v}}_{D}$ and ${\overrightarrow{v}}_{S}$:

8 $${\overrightarrow{v}}_{D}=-\frac{1-D}{\tau }\overrightarrow{\nabla }(\frac{\partial {\chi }_{D}}{\partial D}-{\varepsilon }^{2}\Delta D);{\overrightarrow{v}}_{S}=-\frac{D}{1-D}{\overrightarrow{v}}_{D}$$

From Eq. (8), we can define the cell-cell adhesion in units of *τ* which gives a length unit *l*_0_ as the square root of the cell-cell adhesion multiplied by *t*_0_, the time unit, so hereafter all quantities will be dimensionless. For simplicity, we do not change the notations. Different options can be made for *χ*_*D*_ but must respect physical objectives such as balance of the stresses at the tumor border, itself defined by a border density *D*_*b*_ although this condition assumes no density gradient at the border. In addition, at low densities, *χ* is close to zero, being negative and strongly increases for growing density above *D*_*b*_. A standard choice is the Cahn-Hilliard potential,(see Appendix E) but here, by reason of numerical convergence the potential introduced by Byrne and Preziosi^41,42^ has been preferred. Its derivative reads:

9 $$\frac{\partial {\chi }_{D}}{\partial D}={\alpha }_{D}{D}^{\Lambda }\frac{(D-{D}_{b})}{(1-D)}$$

*α*_*D*_, *D*_*B*_ and Λ being positive constants. *D*_*b*_ is smaller than 1.

### Heterogeneity under tumor growth

Adopting the same strategy as before, we extend the stability analysis of each dynamically stable fixed point, ${ {\mathcal F} }_{1}$ and ${ {\mathcal F} }_{2}$, to spatially periodic perturbations such that each cell or chemical density becomes *ϕ*(*x*, *t*) = *ϕ*_*homo*_ + (*δϕ*)*e*^*λt*^cos(*kx*). Each Jacobian (Eqs (5) and (41)) is then dependent on *k*^2^ and the characteristic polynomial $P(\lambda )=\mathop{\sum }\limits_{j=0}^{3}\,{\lambda }^{j}{C}_{j}({k}^{2})$ which fixes the eigenvalues can be found in the Appendix F. Static and periodic patterns are found for *λ* = *dλ*/*dk* = 0 which imposes that *C*_0_ = *dC*_0_(*k*)/*dk* = 0 as well.

**Figure 3:**
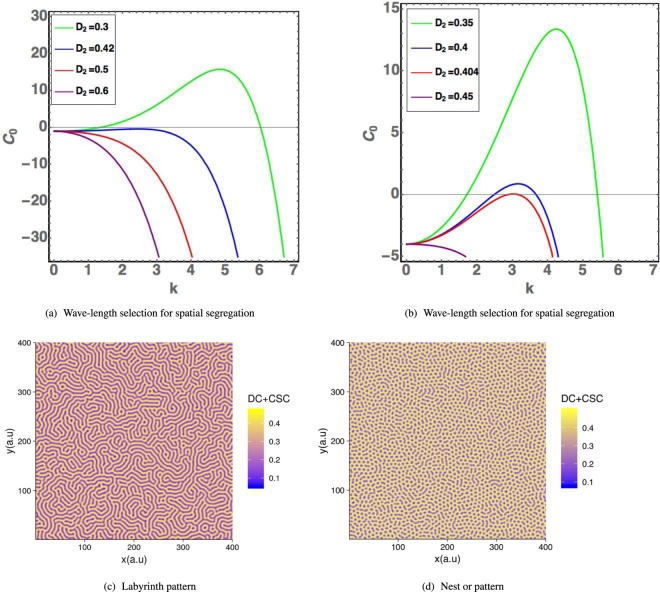
Cell repartition study in the vicinity of fixed points ${ {\mathcal F} }_{2}$ in the domain of dynamical stability (see Fig. (1b)). In (**a,b**), results of the linear stability analysis with variation of the wave number *k* revealed by *C*_0_. In (**a**), the dynamical parameters are: ${ {\mathcal F} }_{2}$ = (0.2*D*_2_, *D*_2_, 0,0.2), with *α* = 1, *D*_*a*_ = *D*_*m*_ = 1 and *B* = −5. in (**b**) the same parameter but with *B* = −20 (see Eq. (16) of the Appendix C). The Byrne-Preziozi potential^41,42^ is selected with the following values *α*_*D*_ = 2, *m* = 1, *D*_*b*_ = 0.6 and *ε*_*D*_ = 0.1 in Eq. (9). Phase separation of tumor cell CSC or DC with the surrounding tissue. In (c): Labyrinth-like pattern for {*d*, *α*} = {0.2, 1} and consequentially {*D*_2_, *S*_2_} ~ {0.23, 0.045}. (**d**): Honeycomb-like pattern for {*d*,*α*} = {0.15,0.8} and consequentially {*D*_2_, *S*_2_} ~ {0.4, 0.06}.

**Figure 4:**
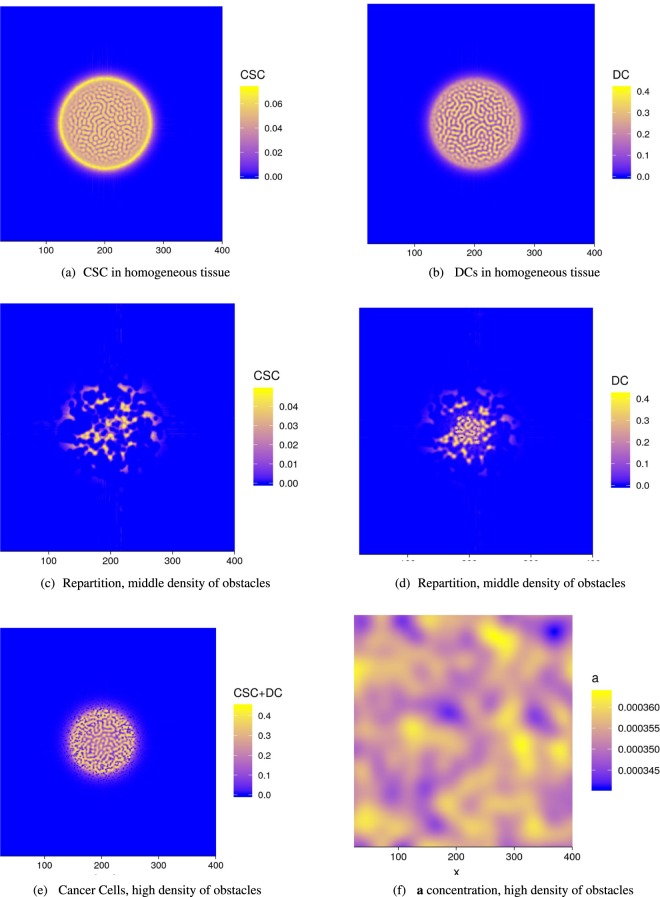
Growing tumors in different tissues and chemical conditions: (**a,b**) in a homogeneous tissue, represented by a standard grid in the simulations, (**c,d**) in a tissue with 2% of randomly distributed obstacles, (**e**) in a tissue with 5% of randomly distributed obstacles. (**f**) Activator **a** density in a medium with high density of obstacles. All patterns are scaled by the length unit *l*_0_, square root of the ratio between the cell-cell adhesion density and the friction coefficient times the time unit so *l*_0_ = (*t*_0_*χ*/*τ*)^1/2^.

**Figure 5:**
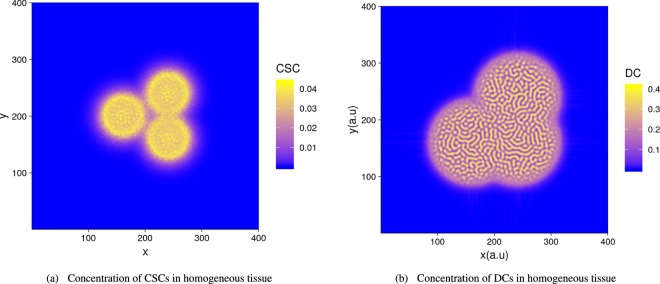
Population of cancer stem cells (**a**) and differentiated cancer cells (**b**) in growing tumors starting from three initial equidistant and separated seeds. Even for primary tumors (non metastatic), it is not rare that several tumors can be found in the same neighborhood.

At linear order, advection does not play any role for ${ {\mathcal F} }_{1}$ since *S*_1_ = *D*_1_ = 0, and the diffusion of chemicals being stabilizing, this fixed point remains stable leading to a homogeneous tumor. As predicted by the linear stability analysis explained in Appendix F and shown in Fig. (3a,b), there exist patterns for ${ {\mathcal F} }_{2}$. The selected wavenumber of the spatial pattern is found in the neighborhood of ${k}_{c}^{2}\sim -\,1/(2{e}^{2})\chi ^{\prime\prime}$, so for *D*_2_ ~ 0.44 according to our choice given by Eq. (9). One can observe that low values of *D*_2_ (i.e. below 0.44) make segregation more likely to occur. Patterns in a square are shown in Fig. (3c,d) and in Fig. (4) in a growing tumor. Notice that in the vicinity of the selected *D*_2_ values, the numerical patterns exhibit either unstable labyrinths or dots. Dots may represent the nests (clinical vocabulary) observed in many tumors. Assuming the soft tissue to be homogeneous, the tumoral seed develops with a phase segregation inside, both for CSCs and DCs, (see Fig.(4a,b)). Surprisingly, despite the interior patterning, the contour remains rather well defined with no contour instability except when obstacles are introduced. With a small density of random obstacles, (see Fig. (4c,d)) the interface is tortuous but in (e) it surprisingly recovers regularity at a high density. CSCs are more located at border except the case of low level of obstacles. This CSCs concentration at the border of tumors is also observed in mice after injection of human CSCS^43^. The fact that CSC have a higher density at the border also indicates that they are good candidate for metastasis. Finally, in Fig. (5), 3 seeds are introduced to mimic more closely the clinical reality. Details on the numerics are given in Section (Methods), however one must mention that modelling of growing tumors is numerically much more challenging when not achieved in a square with periodic boundary conditions.

## Discussion and Conclusion

In this paper, we presented, analyzed and simulated a simple multiphase model for tumor initiation driven by CSC cells. We focused both on the differentiation process of cancer stem cells and on the de-differentiation of differentiated cancer cells within the tumor. We analyzed the effect of chemical activators responsible for these processes and the possible therapeutic strategies for tumor extinction. Our conclusion is that, in this situation, the most effective strategies are the ones that target chemical activators rather than cells themselves, due to a vicious feedback loop between CSCs and DCs. New therapeutics emerge in this direction^29,44,45^. With numerical simulations, we understood how modifications of the environment, in which the tumor expands, can abruptly change its long time behavior. In the future, a clear understanding of the mechanical and physical properties of CSCs can improve the model, especially when considering adhesion energy, elastic deformation and cell-cell interaction^41,46^. In this spirit, a more complicated multiphase model can be constructed, taking into account immune cells, the host cells and their interaction with tumor cells. Because of a lack of information, the existence of niches is dogged here: they may be at the origin of localized sources of *D* cells inside the tumor and would require separation between mobile and static DC populations. Our model can be extended to other cellular cell types which can be reprogrammed in cancer initiated cells^47^ or in cancer stem cells after responses to therapeutics^48^. From the theoretical viewpoint, we have treated here an ecological model where the competition between species depends on a singular tiny sub-population. It will apply probably to other communities than the cancerous cells, opening future directions in nonlinear physics.

## Methods

**Figure 6:**
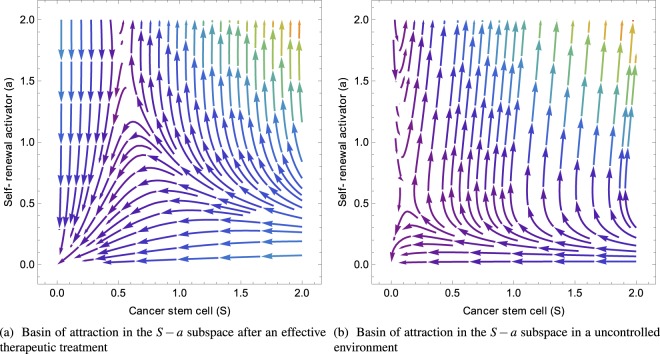
(**a**) Basin of attraction for tumor extinction in case of controlled chemical feedback ({*β*, *α*, *η*, *ψ*} = {3, 1, 1, 1}). (**b**) The basin of attraction of tumor extinction shrinks as the chemicals are less controlled, making the extinction less likely to occur ({*β*, *α*, *η*, *ψ*} = {5, 0.2, 2, 2}). In (**a**), an initial concentration of CSCs will undergo regression and so DCs for every meaningful value of chemicals concentrations. In (**b**), the shrinkage of the basin of attraction for tumour regression may lead to an invasive colony of cancer cells, even in case of a small initial concentration.

The system corresponding to Fig. (6) was numerically solved with the XMDS2 software package^49^. We used an adaptive Runge-Kutta of fourth-fifth order with a tolerance 10^−5^. All spatial operators were evaluated via spectral methods. The simulations were performed on a (400 × 400) grid with a space discretization of *dx* = 10^−1^ and with periodic boundary conditions for Fig. (3) which shows the emergence of different patterns with respect to the fixed point values ${ {\mathcal F} }_{2}$. The most relevant parameters *d* and *α* were varied, keeping fixed in the following the ensemble {*η*, *ψ*, *q*_0_, *m*_0_, *σ*, *β*, *γ*} = {1, 1, 1, 0.5, 0.05, 1, 1} and {*ε*_*D*_, *ε*_*S*_, *ε*_*C*_, Λ, *D*_*b*_, *α*_*D*_, *D*_*m*_, *D*_*a*_} = {0.1, 0, 0, 2, 0.6, 2, 1, 1}. In Fig. (3c) different choices for chemical degradation rates and cell death result in labyrinth-like patterns for both *S* and *D*. In Fig. (3d), upon increasing the value ${ {\mathcal F} }_{2}$, a honeycomb-like pattern emerges. The origin of the different patterns may be due to the highest value of *D*_2_ which increases the effective strength of the cell-cell DCs attraction given by Eq. (9). In Fig. (4) an initially localized concentration of CSCs and DCs grows in space and time. Numerical simulations were performed for different boxes and initial conditions. In Fig. (4) from a to f, *S*, *D* are initialized as a small and localized population with random perturbations around ${ {\mathcal F} }_{2}$. Similar initial conditions apply for the chemicals, but distributed on the entire grid. Concentration fields for both *S*,*D* are evaluated up to *t* = 30 - the time scale being in unit of differentiation of the CSCs - and plotted against each other. In Fig. (4a,b), CSCs and DCs expand in a homogeneous tissue. In particular, Fig. (4a) highlights the relevance of CSCs in this process. CSCs form a ring of higher concentration with respect to bulk values which effectively drives the tumor. In Fig. (4) from c to f, obstacles are introduced inside the box. Obstacles are tumor regions not accessible to tumor cells. In order to model them, simulations were performed on a grid with randomly distributed holes. Numerically, at each step of the iterative process, we cancel the cell density inside the holes corresponding to obstacles and then we integrate the following discretized equation $\frac{\partial {\varphi }_{i}}{\partial t}=({\Gamma }_{{\varphi }_{i}}-{\nabla }_{i}\cdot {\varphi }_{i}{\overrightarrow{v}}_{{\varphi }_{i}}){O}_{i}$. *O*_*i*_ is a (1,0) binary variable, extracted from a Bernoulli distribution with a different mean: *μ* = 0.98 in Fig. (4c,d) and *μ* = 0.95 in Fig. (4e,f). In Fig. (4c,d) the tumor grows in a tortuous fashion, breaking the cylindrical symmetry. This effect is two-sided: first, the addition of obstacles breaks the circular ring and second, the values of ${ {\mathcal F} }_{2}$ are the same as the honeycomb pattern, giving rise to a strong effective attraction between cells. In Fig. (4e), despite a higher number of holes in the grid, the only relevant effect to the tumor growth is the breakdown of the circular ring of CSCs. The size and form of the tumor are qualitatively unaffected.

Our dynamical model presents 3 fixed points, 2 of them are of special interest since they concern a low concentration of cancerous cells. Their stability and basin of attraction have a crucial importance for treatments: they can induce the complete disappearance of the cancer cells. Each fixed point *F*_*i*_ is defined by 4 concentrations values ${ {\mathcal F} }_{i}$ = (*S*_*i*_, *D*_*i*_, *a*_*i*_, *m*_*i*_) which satisfy the dynamical system Eq. (1), without time dependence. The notation *ϕ*_*j*_ is used for either *S*, *D*, *a* or *m*. Following a very classical strategy of dynamical systems^50,51^, each concentration *ϕ* is perturbed according to ${\tilde{\varphi }}_{j}={\varphi }_{j}+{\delta }_{j}{e}^{{\lambda }_{i}t}$ where *δ*_*j*_ is a small quantity and *λ*_*j*_ the growth rate of the perturbation. The stability is entirely governed by the Jacobians of our system, Eq. (10), having coefficient matrix given by ∂Γ_*k*_/∂*ϕ*_*j*_ where Γ_*k*_ has been introduced to represent *dϕ*_*k*_/*dt*. Being local, the stability analysis must be achieved for each fixed point, independently of the others.

**Figure 7:**
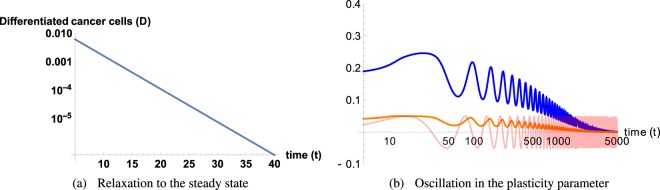
In (**a**) Relaxation to the steady state value of differentiated cell population *D* near the stable point ${ {\mathcal F} }_{1}$ with Runge-Kutta algorithm. Initial concentration {*S*(0), *D*(0), *m*(0), *a*(0)} = {0.1, 0.1, 1.0, 1.0}. With such initial parameters, the system is in the basin of attraction of the first fixed point, so of tumor extinction. The *S* concentration (not represented here) follows the same lines. Time is in the unit of the mitotic rate of *S* cells, see Section (Methods). In (**b**)CSCs and DCs (orange and blue respectively) oscillate with a delay with respect to the oscillating activity *q*_0_(*t*) (light red curve). DCs need CSCs to differentiate, causing a delay in their productions. Likewise, CSCs need DCs to dedifferentiate. Other values of *ω* and *ε* have been tested, without obtaining any substantial difference but a time scale.

## Appendix

### A. Existence and stability of fixed points

The Jacobian matrix, calculated with the densities of each fixed point, is then:

10 $$(\begin{array}{llll}2p-1 & 2{S}_{i}{p}_{D}+q & 2{S}_{i}{p}_{a} & q^{\prime} {D}_{i}\\ 2(1-p) & -\,2{S}_{i}{p}_{D}-(d+q) & -\,2{S}_{i}{p}_{a} & -\,q^{\prime} {D}_{i}\\ \frac{\beta {a}_{i}^{2}}{1+{a}_{i}} & 0 & \beta {S}_{i}\frac{{a}_{i}({a}_{i}+2)}{{({a}_{i}+1)}^{2}}-\alpha  & 0\\ -\,\frac{\gamma }{{S}_{0}}{e}^{-\frac{{S}_{i}}{{S}_{0}}} & 0 & 0 & -\,\alpha \end{array})$$

where:

11 $$\{\begin{array}{rcl}{p}_{D} & = & \frac{\partial p(D,a)}{\partial D}=\frac{-\,\eta \psi a}{(1+\eta a){(1+\psi D)}^{2}}\le 0\\ {p}_{a} & = & \frac{\partial p(D,a)}{\partial a}=\frac{\eta }{{(1+\eta a)}^{2}(1+\psi D)} > 0\\ q^{\prime}  & = & \frac{dq(m)}{dm}=\frac{1}{2\sigma }{\cosh }^{-2}(\frac{m-{m}_{0}}{\sigma })\\  & = & \frac{2q(m)}{\sigma }(1-q(m)/{q}_{0}) > 0\end{array}$$

### B. Stability of the two first fixed points: ${ {\mathcal F} }_{1}$ and ${ {\mathcal F} }_{2}$

#### B.1 Stability of ${ {\mathcal F} }_{1}$ = (0, 0, 0, *m*_1_)

This fixed point corresponds to complete eradication of the tumor. The Jacobian reads:

12 $${J}_{1}=(\begin{array}{llll}-1 & q({m}_{1}) & 0 & 0\\ 2 & -(d+q({m}_{1})) & 0 & 0\\ 0 & 0 & -\alpha  & 0\\ -\frac{\gamma }{{S}_{0}} & 0 & 0 & -\alpha \end{array})$$

For this setting, the eigenvalues are:

13 $$\{\begin{array}{rcl}{\lambda }_{1}^{(1)} & = & {\lambda }_{1}^{(2)}=-\alpha \\ {\lambda }_{1}^{(3)} & = & \frac{1}{2}(-(1+d+q({m}_{1}))-{S}_{q})\\ {\lambda }_{1}^{(4)} & = & \frac{1}{2}(-(1+d+q({m}_{1}))+{S}_{q})\\ {S}_{q} & = & \sqrt{{(1+d+q({m}_{1}))}^{2}-4(d-q({m}_{1}))}\end{array}$$

#### B.2 Stability of ${ {\mathcal F} }_{2}$ = (***S***_2_, ***D***_2_, 0, *m*_2_)

with *D*_2_ = *S*_2_/*d* and *S*_2_ = −*S*_0_*Log*[(*αm*_2_)/*γ*]. After straightforward simplifications, the Jacobian for this fixed point is then:

14 $$\begin{array}{l}{J}_{2}=(\begin{array}{llll}-\,1 & d & \frac{2{S}_{2}\eta }{1+\psi {D}_{2}} & \frac{2d{D}_{2}}{\sigma }(1-\frac{d}{{q}_{0}})\\ 2 & -\,2d & -\frac{2{S}_{2}\eta }{1+\psi {D}_{2}} & -\,\frac{2d{D}_{2}}{\sigma }(1-\frac{d}{{q}_{0}})\\ 0 & 0 & -\,\alpha  & 0\\ -\,\frac{\alpha }{{S}_{0}}{m}_{2} & 0 & 0 & -\,\alpha \end{array})\end{array}$$

One eigenvalue obviously being *λ*_1_ = −*α*, the three others are determined by solving the third order characteristic polynomial:

15 $${\lambda }_{2}^{3}+{\lambda }_{2}^{2}(\alpha +1+2d)+{\lambda }_{2}(\alpha (1+2d)-B)-Bd=0,$$

where $B=\frac{2\alpha }{\sigma }(1-\frac{d}{{q}_{0}}){m}_{2}\,\log (\frac{\alpha }{\gamma }{m}_{2})$. Since *d* < *q*_0_, as a condition for ${ {\mathcal F} }_{2}$ to exist, and *m*_2_ < *γ*/*α*, *B* is negative and all coefficients in the characteristic polynomial are then positive. In the case of a polynomial written as *λ*^3^ + *pλ*^2^ + *qλ* + *r*, with all coefficients *p*, *q*, *r* being positive, the condition for negative real parts of the 3 roots is simply *pq* > *r* according to the Routh-Hurwitz result^52^. This condition is always satisfied in our case. The second fixed point is then always stable and is an attractor. With the Sturm Theorem, it is possible to prove that there exists only one negative real eigenvalue and a pair of two complex conjugates eigenvalues with negative real part. Let us proceed now to the analysis of the last fixed point.

### C. Existence and stability analysis of the fixed point ${ {\mathcal F} }_{3}$

The existence of the third fixed point corresponds to *q*(*m*_3_) ~ 0 and *p* ~ 1/2, which allows to get an approximate value of *a*_3_ by solving *p*(*a*_3_, *D*_3_) = 1/2. It follows an algebraic second order equation for *a*_3_:

16 $${a}_{3}^{2}(1-{\psi }_{0})\eta -{a}_{3}(1+{\psi }_{0}(1+\eta ))-{\psi }_{0}=0$$

where *ψ*_0_ = *ψα*/(*dβ*). A unique positive root can be found:

17 $${a}_{3}=\frac{1+{\psi }_{0}(1+\eta )}{2\eta (1-{\psi }_{0})}\{1+\sqrt{1+\frac{4{\psi }_{0}\eta (1-{\psi }_{0})}{{(1+{\psi }_{0}(1+\eta ))}^{2}}}\}$$

when *ψ*_0_ < 1. From Eq. (16), we can deduce also that:

18 $${\psi }_{0}=\frac{{a}_{3}(\,-\,1+\eta {a}_{3})}{(1+{a}_{3})(1+\eta {a}_{3})}$$

suggesting that *a*_3_ > 1/*η* since *ψ*_0_ must be positive. So finally we get for our third fixed point:

19 $${{\mathscr{F}}}_{3}=(\frac{\alpha }{\beta }\frac{(1+{a}_{3})}{{a}_{3}},\frac{\alpha }{\beta d}\frac{(1+{a}_{3})}{{a}_{3}},{a}_{3},\frac{\gamma }{\alpha }{e}^{-{S}_{3}/{S}_{0}})$$

The treatment is more difficult for this fixed point if we do not make the appropriate approximations which lead to its condition of existence. Indeed, without approximation, from Eq. (10), the characteristic polynomial is expected to be of 4th order, then requiring a numerical treatment. Assuming that *ψ*_0_ is smaller than 1 but not too close to 1, we can consider that ${e}^{-{S}_{3}/{S}_{0}} \sim 0$, since *S*_0_ is a tiny quantity compared to ${S}_{3}=\frac{\alpha }{\beta }\frac{(1+{a}_{3})}{{a}_{3}}$. In addition, *q*(*m*_3_) ~ 0 and *p* ~ 1/2. This simplifies the Jacobian which reads:

20 $$\begin{array}{l}{J}_{3} \sim (\begin{array}{llll}0 & -\,d\frac{{\tau }_{0}{\psi }_{0}}{2\eta } & {\tau }_{0}\frac{\alpha }{\beta {(1+\eta {a}_{3})}^{2}} & q^{\prime} {D}_{3}\\ 1 & d(\frac{{\tau }_{0}{\psi }_{0}}{2\eta }-1) & -\,{\tau }_{0}\frac{\alpha }{\beta {(1+\eta {a}_{3})}^{2}} & -\,q^{\prime} {D}_{3}\\ \frac{\beta {a}_{3}^{2}}{1+{a}_{3}} & 0 & \frac{\alpha }{(1+{a}_{3})} & 0\\ 0 & 0 & 0 & -\,\alpha \end{array})\end{array}$$

where *τ*_0_ = (1 + *a*_3_)(1 + *ηa*_3_)/*a*_3_^2^ and *q*′ = *q*′(*m*_3_). We can conclude that, there is at least a negative eigenvalue −*α*. The characteristic polynomial for the eigenvalues of the third fixed point, independently of *λ*_0_ = −*α* reads:

21 $${\lambda }_{3}^{3}+{C}_{2}{\lambda }_{3}^{2}+{C}_{1}{\lambda }_{3}+{C}_{0}=0$$

where

22 $$\{\begin{array}{rcl}{C}_{2} & = & d-\frac{\alpha }{1+{a}_{3}}-d{\tau }_{0}\frac{{\psi }_{0}}{2\eta }\\ {C}_{1} & = & -\alpha (\frac{1}{1+\eta {a}_{3}}+\frac{d}{1+{a}_{3}})+\frac{d{\psi }_{0}}{2{a}_{3}^{2}\eta }(1+{a}_{3}+\alpha )(1+{a}_{3}\eta )\\ {C}_{0} & = & -\alpha d(\frac{1}{1+{a}_{3}\eta }+\frac{(1+{a}_{3}\eta ){\psi }_{0}}{2{a}_{3}^{2}\eta })\end{array}$$

*C*_0_ represents the opposite of the product of the 3 roots. Since *C*_0_ is negative, there is at least always one positive eigenvalue. For a first set of parameters: {*α*, *β*, *d*, *ψ*, *η*} = {1, 3, 2, 1, 1}, so *ψ*_0_ = 1/6, the corresponding characteristic polynomial is then:

23 $$P(\lambda )={\lambda }^{3}+1.21446{\lambda }^{2}-0.53327\lambda -0.889899$$

with roots:

$$-1.01129-0.280182i,\,-\,1.01129+0.280182i,\,0.808115$$

A second choice: {*α*, *β*, *d*, *ψ*, *η*} = {0.5, 5, 0.2, 0.5, 1}, giving *ψ*_0_ = 0.25, has the following characteristic polynomial:

24 $$P(\lambda )={\lambda }^{3}-0.0120835{\lambda }^{2}-0.128109\lambda -0.0401924$$

with the following roots:

$$-0.228239-0.183536i,-\,0.228239+0.183536i,\,0.468561$$

which also indicates a saddle point. However, due to the number of independent parameters, the study of the roots of the characteristic polynomial of third order remains cumbersome. Indeed, the product of the 3 roots being positive indicates that at least one real eigenvalue is positive, suggesting that the third fixed point is always a saddle point, once it exists (*ψ*_0_ < 1), independently of all parameters.

### D. Numerical investigation of the dynamical system

#### D.1 First case: *d* > *q*_0_*q*(*m*_1_) ~ *q*_0_

We select numerical values of parameters such that both ${ {\mathcal F} }_{1}$ and ${ {\mathcal F} }_{3}$ exist, the first one being stable but in the neighborhood of ${ {\mathcal F} }_{3}$ which is unstable. In this case and as shown in Fig. (1b), ${ {\mathcal F} }_{1}$ is the only attractive fixed point which gives the optimistic issue of the full tumor regression. However, ${ {\mathcal F} }_{3}$ can possibly prevent the cancer regression, even in such a favorable situation. We numerically focus on the set {*α*, *β*, *d*, *ψ*, *η*} = {1, 3, 2, 1, 1}, giving *ψ*_0_ = *αψ*/(*dβ*) = 1/6 which is smaller than 1 as required for the existence of ${ {\mathcal F} }_{3}$. Choosing {*γ*, *m*_0_, *σ*, *S*_0_, *q*_0_} = {1, 0.5, 0.05, 0.038, 1}, it is found ${ {\mathcal F} }_{1}$ = (0, 0, 0, 1), stable since *d* > *q*_0_ and from Eq. (19), we numerically deduce:

25 $${F}_{3}=\{{S}_{3},{D}_{3},{a}_{3},{m}_{3}\}=\{0.53,0.26,1.72,{10}^{-6}\}$$

The value of *σ* is chosen to mimic conveniently a Heaviside function for *q*(*m*). The computed eigenvalues of the Jacobian from Eq. (10) corresponds perfectly to Eq. (23) which validates our approximated analysis of the stability of the third fixed point. It also proves that, for the determination of the eigenvalues which fixes the dynamics, the only pertinent parameters are {*α*, *β*, *d*, *ψ*, *η*} and that the precise values of {*S*_0_, *σ*, *γ*} have much less importance. For this particular choice, we have 3 directions of attraction, 2 being spirals and one being a repulsive direction, see Fig. (2d). As shown analytically, this is a saddle point. In Fig. (2a,b), the population of DC and CSC cells are given as a function of time in a log-scale where a time-scale relaxation can be estimated and compared to the smallest (in absolute value) growth rate predicted by Eq. (13), *λ*_1_^(4)^ ~ −0.536, see Fig. (7a).

#### D.2 Second case: *d* < *q*_0_

Here, ${ {\mathcal F} }_{1}$ will be repulsive, while ${ {\mathcal F} }_{2}$ will be attractive and ${ {\mathcal F} }_{3}$ is a saddle fixed point (c.f Fig.(1b)). The following parameters are selected: {*α*, *β*, *d*, *ψ*, *η*} = {0.5, 5, 0.2, 0.5, 1}, the other parameters being fixed as: {*γ*, *m*_0_, *σ*, *S*_0_, *q*_0_} = {1, 0.05, 0.038, 1}. For ${ {\mathcal F} }_{3}$ = {*S*_3_, *D*_3_, *a*_3_, *m*_3_} = {0.052, 0.26, 2.147, 0.51}. The eigenvalues for ${ {\mathcal F} }_{3}$ are calculated with Eq. (10) and gives the same results as Eq. (24). The situation looks similar to the previous case but the main and important difference relies on the existence of two repulsive fixed points, ${ {\mathcal F} }_{1}$ and ${ {\mathcal F} }_{3}$ (see Fig. (1b)). As shown in Fig. (2b), one striking feature is the overshoot of the dynamical variables induced by the feedback activator *m*. The tumor will not go under regression most of the time, although ${ {\mathcal F} }_{2}$ is an attractive fixed point, Fig. (2c). Moreover even when the system reaches a steady state, it can maintain a high level of differentiated cells if *d* is small. The tumor will not proliferate but may stabilize at dangerous levels, which is not an optimistic issue in practice.

#### Time dependent perturbation of the dynamical system: Stochasticity of the chemicals

We proceed by investigating the dynamics of the system numerically. We perform numerical simulations of Eq. (1) when *q*_0_(*t*) is an explicit time-dependent function. Of particular interest is the case where *q*_0_(*t*) oscillates around *d*, in the area of the bisector. In this regime, we would expect the system to exhibit an oscillatory behavior, due to the switching in the stability of the fixed points: ${ {\mathcal F} }_{1}$ <=> ${ {\mathcal F} }_{2}$. In particular, ${ {\mathcal F} }_{1}$ will change from a stable fixed point (tumor regression) to an unstable fixed point. The concentration of cells will then be pushed away from its decreasing value when at a particular time *t*_1_, *q*_0_(*t*_1_) > *d* and pushed back toward regression when *q*_0_(*t*_2_) < *d*. In Fig. (7), we show numerical results for *q*_0_ = *d* + *f*(*t*), *f*(*t*) being a periodic function with period *T*. Despite the symmetry around *d*, the system spending equal time in the different regions of the phase space, the tumor tends to go under regression at long times. Intuitively, the system relaxes to the different attractors in each regions (${ {\mathcal F} }_{1}$ and ${ {\mathcal F} }_{2}$) with a time scale governed by the slowest decaying mode. We compare the slowest decaying modes in the two extreme cases, *q*_0_^(*max*)^ = *d* + *f*_*max*_ and *q*_0_^(*min*)^ = *d* + *f*_*min*_. In the first case, being *q*_0_^(*max*)^ > *d*, the stable fixed point is ${ {\mathcal F} }_{2}$, with slowest decaying mode: *λ* = max*λ*_*i*_ ~ −0.2 and associated time-scale $\tau =\frac{1}{|\lambda |} \sim 5$. In the second case, *q*_0_^(*min*)^ < *d*, the stable fixed point is ${ {\mathcal F} }_{1}$, with slowest decaying mode: *λ* = max*λ*_*i*_ ~ −0.04 and associated time-scale $\tau =\frac{1}{|\lambda |} \sim 25$. We would expect a faster relaxation toward ${ {\mathcal F} }_{2}$ than ${ {\mathcal F} }_{1}$, but this is not observed in Fig. (7). In particular, when the Jacobian matrices are not normal the instantaneous response -which determines the dynamic for a time varying *q*_0_(*t*)- is given by the eigenvalues of its hermitian part: *H*_*J*_ = (*J* + *J*^*T*^)/2^53^. The hermitian matrix, *H*_*J*_, has positive eigenvalues in both scenarios, making an analytical understanding of Fig. (7) challenging. Considering *a* and *m* as a stochastic variables, our preliminary study indicates that the noise stabilizes ${ {\mathcal F} }_{1}$ which seems to be selected rather than ${ {\mathcal F} }_{2}$ even in area where ${ {\mathcal F} }_{2}$ is stable without noise. This is especially verified above the bisector. Below the bisector, the answer is more complex but either ${ {\mathcal F} }_{1}$ or ${ {\mathcal F} }_{2}$ are selected. A more extensive study of stochasticity can be found in^54^. The results obtained for sinusoidal perturbations in the asymptotic may be due to the choice of initial data.

### E. Spatio-dynamical equations deduced from the Rayleighian principle

The Rayleighian ${\mathcal R}$ represents the energy variation per unit time for a system whose dynamics results only from dissipation, as for Stokes flow. When inertia is discarded, a variational principle of extremum of dissipation applies and the constraints acting on the system are included via Lagrange parameters *Q*_*i*_. It reads:

26 $${\mathscr{R}}=\frac{{\rm{d}}{\mathscr{E}}}{{\rm{d}}t}+{\mathscr{D}}-\mathop{\int }\limits_{\Omega (t)}\,Q{\rm{\nabla }}({\varphi }_{i}{\overrightarrow{v}}_{i})d\Omega $$

${\mathcal E}$ is the interaction energy between phases, the viscous dissipation ${\mathscr{D}}$ includes both interphase friction and viscosity and Ω is the tumor volume at time *t*. Here, only the constraint of global incompressibility is applied (via the function *Q*). Let us define more precisely each term in our case. The energy density ${\mathcal E}$ is the sum of the energy of each phase *S*, *D* and *C*, the energy of interaction between phases and a penalty imposed to strong density gradient variations: The energy variation is then:

27 $$ {\mathcal E} =\mathop{\sum }\limits_{i=1}^{3}\,\mathop{\int }\limits_{\Omega (t)}\,({\chi }_{{\varphi }_{i}}-\frac{1}{2}{\varepsilon }_{i}^{2}\overrightarrow{\nabla }{\varphi }_{i}^{2})$$

where *χ*_*ϕi*_ is the cell-cell interaction of *i* with all other phases. In practice, in our case, *χ*_*ϕi*_ being scaled by *ϕ*_*i*_*ϕ*_*j*_, the sum reduces to *χ*_*D*_ as soon as the *C* phase is completely inert. The viscous dissipation rate reads:

28 $${\mathscr{D}}=\mathop{\sum }\limits_{i=1}^{3}\,\mathop{\int }\limits_{\Omega (t)}\,\frac{1}{4}\mathop{\sum }\limits_{j=1}^{3}\,\tau {\varphi }_{i}{\varphi }_{j}\cdot {({\overrightarrow{v}}_{i}-{\overrightarrow{v}}_{j})}^{2}+\frac{\mu }{2}{\varphi }_{i}{(\nabla {\overrightarrow{v}}_{i})}^{2}$$

where *τ* is a mobility coefficient and *μ* the viscosity. The time variation of the free-energy is decomposed in two terms for a growing domain Ω(*t*): a surface (resp. a volume) integral calculated on Ω and a line (resp. a surface) integral along ∂Ω(*t*).

29 $$\frac{{\rm{d}} {\mathcal E} }{{\rm{d}}t}=\mathop{\int }\limits_{\Omega (t)}\,\frac{\partial  {\mathcal E} }{\partial t}d\Omega +\mathop{\int }\limits_{\partial \Omega }\, {\mathcal E} (\overrightarrow{N}\cdot {\overrightarrow{v}}_{\omega })dl$$

where ${\overrightarrow{v}}_{\omega }$ is the interface velocity. Time dependence of *ϕ* is given by:

30 $$\frac{\partial {\varphi }_{i}}{\partial t}={\Gamma }_{{\varphi }_{i}}-\nabla ({\varphi }_{i}{\overrightarrow{v}}_{{\varphi }_{i}})$$

#### E.1 Inside the tumor

Let us first consider the dynamical equation inside the tumor. Considering an extremum of the Rayleighian with respect to ${\overrightarrow{v}}_{S}$, ${\overrightarrow{v}}_{D}$ and ${\overrightarrow{v}}_{C}$ gives the following 3 relationships:

31 $$\tau SD({\overrightarrow{v}}_{S}-{\overrightarrow{v}}_{D})+\tau SC({\overrightarrow{v}}_{S}-{\overrightarrow{v}}_{C})-\mu S\Delta {\overrightarrow{v}}_{S}+S\nabla (\frac{\partial {\bar{\chi }}_{S}}{\partial S}-{\varepsilon }_{S}^{2}\Delta S)+S\nabla Q=0$$

32 $$\tau SD({\overrightarrow{v}}_{D}-{\overrightarrow{v}}_{S})+\tau DC({\overrightarrow{v}}_{D}-{\overrightarrow{v}}_{C})-\mu D\Delta {\overrightarrow{v}}_{D}+D\nabla (\frac{\partial {\bar{\chi }}_{D}}{\partial D}-{\varepsilon }_{D}^{2}\Delta D)+D\nabla Q=0$$

33 $$\tau CS({\overrightarrow{v}}_{C}-{\overrightarrow{v}}_{S})+\tau CD({\overrightarrow{v}}_{C}-{\overrightarrow{v}}_{D})-\mu C\Delta {\overrightarrow{v}}_{C}-{\varepsilon }_{C}^{2}C\nabla \Delta C+C\nabla Q=0$$

where ${\bar{\chi }}_{S}={\chi }_{S}+{\chi }_{SD}$ and ${\bar{\chi }}_{D}={\chi }_{D}+{\chi }_{SD}$. Summing the three equations of Eq. (31) gives $\nabla Q$:

34 $$\nabla Q={\varepsilon }_{C}^{2}C\nabla \Delta C-S\nabla (\frac{\partial {\bar{\chi }}_{S}}{\partial S}-{\varepsilon }_{S}^{2}\Delta S)-D\nabla (\frac{\partial {\bar{\chi }}_{D}}{\partial D}-{\varepsilon }_{D}^{2}\Delta D)$$

Finally, assuming *μ* → 0 we derive:

35 $$\tau {\overrightarrow{v}}_{S}=D\nabla (\frac{\partial {\bar{\chi }}_{D}}{\partial D}-{\varepsilon }_{D}^{2}\Delta D)+{\varepsilon }_{C}^{2}C\nabla \Delta C-(1-S)\nabla (\frac{\partial {\bar{\chi }}_{S}}{\partial S}-{\varepsilon }_{S}^{2}\Delta S)$$

36 $$\tau {\overrightarrow{v}}_{D}=S\nabla (\frac{\partial {\bar{\chi }}_{S}}{\partial S}-{\varepsilon }_{S}^{2}\Delta S)+{\varepsilon }_{C}^{2}C\nabla \Delta C-(1-D)\nabla (\frac{\partial {\bar{\chi }}_{D}}{\partial D}-{\varepsilon }_{D}^{2}\Delta D)$$

Classical choices for the free energy are inspired by the classical Cahn-Hilliard potential:

37 $${\chi }_{D}=-{\alpha }_{D}\frac{{D}^{2}}{2}(1-\frac{{D}^{2}}{{D}_{b}^{2}});{\chi }_{S}=-{\alpha }_{S}\frac{{S}^{2}}{2}(1-\frac{{S}^{2}}{{S}_{b}^{2}})$$

while

38 $${\chi }_{SD}=-{\alpha }_{SD}\frac{SD}{2}((1-\frac{D}{{D}_{b}})(1-\frac{S}{{S}_{b}}))$$

which allows to determine order of magnitudes of velocities in the realistic limit: *D* ≫ *S*. Indeed, ∂*χ*_*D*_/∂*D* ~ *α*_*D*_*D* while ∂*χ*_*S*_/∂*S* ~ *α*_*S*_*S* and ∂*χ*_*DS*_/∂*S* ~ *α*_*DS*_*D* and  *χ*_*DS*_/∂*D* ~ *α*_*S*_. It shows that we can keep only *χ*_*D*_ and it reads:

39 $${\overrightarrow{v}}_{D}=-\frac{1-D}{\tau }\overrightarrow{\nabla }\{\frac{\partial {\chi }_{D}}{\partial D}-{\varepsilon }^{2}\Delta D\};{\overrightarrow{v}}_{S}=-\frac{D}{1-D}{\overrightarrow{v}}_{D}$$

In this case, both velocities are opposite in direction with quite the same order of magnitude. If *α*_*DS*_ ⋙ *α*_*d*_ and *α*_*s*_, then both velocities have the same direction but the stem cells velocity dominates.

#### E.2 Boundary conditions at the tumor border

First of all, it must be emphasized that our treatment is rather general and can be applied to any cell-cell interaction potential. In our simulations, no boundary conditions are required since our simulations correspond to an initial value problem and we do not take into account the exterior of the tumor assumed like a gel or a liquid. However, for completeness and future works, we briefly remind how they are determined from the variational process. They are helpful for selecting the densities *D*_*b*_ and *S*_*b*_ which enter the free energies Eq. (37). In few cases, as for melanoma, the cancerous cell densities at border may be measured and the experimental values are chosen for *D*_*b*_ and *S*_*b*_. At the frontier, the interface velocity is the averaged velocity of the cancerous cells: $\partial \dot{\Omega }(t)=(S{\overrightarrow{v}}_{S}+D{\overrightarrow{v}}_{D})/(S+D)$ if there is no phase separation. Indeed, it is possible that the mixing is fully inhomogeneous and that the phases split up from each other. Considering the terms which have been left by integration by part in the variation with respect to ${\overrightarrow{v}}_{D}$, we are faced with a contour integral:

40 $$\mathop{\int }\limits_{\partial \Omega (t)}\,(\frac{D}{S+D}E-D\frac{\partial {\bar{\chi }}_{D}}{\partial D}-QD)\overrightarrow{N}\cdot (\delta {\overrightarrow{v}}_{D})dl=0$$

where the first term comes from the contour integral of Eq. (29) while the last two terms come from the first integral of the same relation, once integrated by parts using Eqs (26) and (29). A similar equation is verified for *S* cells. Then we have 2 relations at the border: one gives the Lagrange parameter *Q* and the second, one (among 2) density value.

Assuming that the border is the same for *D* and *S* cells (full mixing, no phase segregation) imposes $\frac{\partial {\bar{\chi }}_{D}}{\partial D}=\frac{\partial {\bar{\chi }}_{S}}{\partial S}$. Choosing the Cahn-Hilliard free energy for *χ*_*D*_ and *χ*_*S*_, (Eq. (37)), with *D* = *D*_*b*_ and *S* = *S*_*b*_ leads to *E* = 0 and $\frac{\partial {\bar{\chi }}_{D}}{\partial D}={a}_{D}{D}_{b}$ while $\frac{\partial {\bar{\chi }}_{S}}{\partial S}={a}_{S}{S}_{b}$. So boundary conditions cannot be checked without the help of boundary layers of different thicknesses for both the *S* and *D* populations. A naive relationship will be found for the thicknesses of both boundary layers:

$${\alpha }_{S}{S}_{b}-{\varepsilon }_{S}^{2}{S}_{b}^{2}/{l}_{S}^{2} \sim {\alpha }_{D}{D}_{b}-{\varepsilon }_{D}^{2}{D}_{b}^{2}/{l}_{D}^{2} \sim Q$$

Having now the full spatio-dynamical equations, we can now investigate the stability analysis.

### F. Stability analysis in the vicinity of dynamically stable fixed points

A spatial perturbation is added to any constituent of our system: *ϕ*(*x*, *t*) = *ϕ*_*homo*_ + *δϕ*(*t*)cos(*kx*). The stability of the first fixed point will not change, for any wave-vector. For the second fixed point ${ {\mathcal F} }_{2}$, the Jacobian will be: *J*_2_^*k*^ = *J*_2_ + *k*^2^/*τδJ*, *J*_2_ has yet been defined in Eq. (14) and *δJ* reads:

41 $$\begin{array}{l}{J}_{2}^{k}=(\begin{array}{lll}{j}_{11} & {j}_{12} & 0\\ {j}_{21} & {j}_{22} & 0\\ 0 & 0 & \tau {D}_{m}\end{array})\end{array}$$

Let us first define:

42 $${G}_{S}={S}_{2}\frac{{\partial }^{2}{\chi }_{S}}{\partial {S}^{2}}{|}_{{S}_{2}};{G}_{D}={D}_{2}\frac{{\partial }^{2}{\chi }_{D}}{\partial {D}^{2}}{|}_{{D}_{2}};\tilde{G}=\frac{{\partial }^{2}{\chi }_{SD}}{\partial {D}_{2}\partial {S}_{2}}$$

Then we derive:

43 $$\begin{array}{rcl}{j}_{11} & = & -(1-{S}_{2}){G}_{S}+{S}_{2}{D}_{2}\tilde{G}\\ {j}_{12} & = & {S}_{2}({G}_{D}-(1-{S}_{2})\tilde{G}-{\varepsilon }^{2}(1-{D}_{2}-{S}_{2}){k}^{2})\\ {j}_{21} & = & {D}_{2}({G}_{S}-(1-{D}_{2})\tilde{G}+{\varepsilon }^{2}{k}^{2}{S}_{2})\\ {j}_{22} & = & -(1-{D}_{2}){G}_{D}+{S}_{2}{D}_{2}\tilde{G}-{\varepsilon }^{2}{k}^{2}(1-{D}_{2}){D}_{2}\end{array}$$

The characteristic polynomial of third order is then transformed into:

44 $${\lambda }^{3}+{\lambda }^{2}{C}_{2}+\lambda {C}_{1}+{C}_{0}=0,$$

with all coefficients being dependent of *k*. This equation accepts 3 roots *λ* and a spatial static instability is obtained for the double condition *C*_0_ = −*λ*_1_*λ*_2_*λ*_3_ = 0 and *dC*_0_/*dk* = 0 which gives us the value of the threshold for instability and the wavelength at threshold. Due to the number of independent parameters, it is more convenient to fix the 3 parameters: *α*, *B*, *d*_0_ which partially define the initial fixed point ${ {\mathcal F} }_{2}$ up to the value of *D*_2_, so all the curves *C*_0_(*k*) will start at the same value and will differ because of *D*_2_. A representation of a spinodal decomposition can be found in the Section (Spatio-Temporal), see Fig. (3c,d)) with the Byrne-Preziosi potential^41,42^.
